# Does stage of illness influence recovery-focused outcomes after psychological treatment in bipolar disorder? A systematic review protocol

**DOI:** 10.1186/s13643-019-1042-4

**Published:** 2019-05-25

**Authors:** Hailey Tremain, Kathryn Fletcher, Jan Scott, Carla McEnery, Michael Berk, Greg Murray

**Affiliations:** 1 Centre for Mental Health, Faculty of Health Arts and Design, Swinburne University, PO Box 218, John St Hawthorn VIC, Melbourne, 3122 Australia; 2 Academic Psychiatry, Institute of Neuroscience, Newcastle University, Newcastle, UK; 3 IMPACT Strategic Research Centre, School of Medicine, Barwon Health, Deakin University, Geelong, VIC Australia; 4 Orygen, The National Centre of Excellence in Youth Mental Health, Parkville, Australia; 5 Centre for Youth Mental Health, The University of Melbourne, Melbourne, Australia; 6 The Department of Psychiatry and the Florey Institute for Neuroscience and Mental Health, the University of Melbourne, Parkville, VIC Australia

**Keywords:** Bipolar disorder, Psychosocial interventions, Psychotherapy, Treatment, Moderator, Clinical staging, Stage of illness, Recovery, Functioning, Quality of life

## Abstract

**Background:**

There is great interest in the possibility that ‘stage of illness’ moderates treatment outcomes in bipolar disorder (BD). Much remains unknown about the construct of stage of illness, but there is evidence that effectiveness of psychosocial interventions may depend on factors that are plausible proxy measures of stage of illness (e.g., number of episodes). To date, reviews of this data have focused solely on clinical outcomes (particularly symptoms and relapse rates), but a range of recovery-focused outcomes (including functioning, cognitive functioning, and quality of life) have been measured in individuals with established BD. The aim of the proposed systematic review is to synthesise existing evidence for plausible proxy measures of stage of illness as moderators of recovery-focused and functional outcomes in psychosocial treatment studies of BD.

**Methods:**

The proposed review will follow PRISMA guidelines; Scopus, PsychINFO, PubMed and Web of Science will be searched for empirical studies of psychosocial interventions used for established (clinical stages 2–4) BD; and findings will be summarised in a narrative synthesis of clinical stage of illness (operationalised in proxy measures identified in existing staging models) as a moderator of recovery-focused and functional outcomes of psychosocial interventions for established bipolar disorder.

**Discussion:**

This review will contribute to the literature by expanding upon previous reviews and potentially inform the psychosocial treatment of established BD. Implications include assisting clinicians, consumers and researchers to identify and select interventions most appropriate to recovery-focused goals based on individuals’ clinical status.

**Systematic review registration:**

PROSPERO CRD42016037868

**Electronic supplementary material:**

The online version of this article (10.1186/s13643-019-1042-4) contains supplementary material, which is available to authorized users.

## Background

A developing body of research suggests that bipolar disorder (BD) may be characterised by progressive changes over time, such that the clinical and functional features experienced differ in those earlier versus later in the course of illness [1–5]. Most importantly, it has been argued that the notion of ‘stage of illness’ has potential to generate long-awaited improvements in outcomes for those with BD, via tailoring of treatments to particular stages of illness [4, 6, 7]. Empirical research into staging of illness in BD is in its infancy, with existing studies limited in important ways. Below, we briefly review what is known about staging of illness in the psychosocial treatment of BD and identify key gaps to be addressed by the proposed systematic review.

### Staging models

Several (largely compatible) clinical staging models have been described to capture the key features of BD within putative stages of illness. These broadly refer to an initial asymptomatic at-risk stage, followed by a stage characterised by non-specific symptoms and then a stage with more specific mood disorder-related, but subsyndromal symptoms. A syndromal stage, usually referred to as clinical stage 2 then follows, wherein mood episodes meet recognised diagnostic criteria and functional impacts begin to emerge, followed by stage 3, where a repeated pattern of recurrences and relapses is common. The final or end stage (stage 4) is characterised by chronicity manifested by persistent or highly recurrent symptoms and progressively severe functional impacts [8, 9]. Berk and colleagues [8] highlight the role of accumulating mood episodes and associated functional impairments. A convergent (yet distinct) model proposed by Kapczinski and colleagues [9] primarily focuses on inter-episode functional and cognitive decrements as BD progresses. This model only addresses post-onset threshold disorder and does not detail the subsyndromal phases of BD.

Models of clinical staging in BD remain largely theoretical: no model has been subjected to systematic empirical testing. Stage of illness is conceptualised as multidimensional and multisystemic, encapsulating more than severity. Severity, characterised by linear and unidimensional changes (increasingly more severe symptoms or more frequent episodes) may represent one factor which changes between stages of illness [10]. However, additional related features are hypothesised to distinguish stages of illness, reflecting progression and extension of disease: medical and psychiatric comorbidities, cognitive decrements, reduced response to treatment and psychosocial sequelae (such as loss of autonomy and social functioning), although the boundaries of stages remain undefined [11]. Such stages could, in future, be distinguished by biomarkers. However, key questions remain around biological markers of this proposed multisystemic, pathophysiological progression and, critically, the proportion of individuals who experience progression through each stage of illness [10, 12]. Nonetheless, the International Society for Bipolar Disorders Taskforce for Staging has concluded that sufficient evidence has accumulated to support the use of staging of illness within established BD (i.e., post-onset or full-threshold disorder), which we will refer to as clinical stages 2–4. Also, the task force notes that such a distinction is likely to be of heuristic value in the treatment of BD [13]. Likewise, data-driven approaches have begun to distinguish broad post-onset groups based on features such as functioning, number of episodes, and illness duration, and these groups show different responses to treatment [5, 14, 15]. There is also some evidence that neuroimaging and cognition vary by stage of illness and emerging evidence of biomarkers that may track illness trajectory [15, 16].

### Benefits of illness staging for bipolar disorder

A validated staging model for bipolar disorder therefore holds significant, but as yet unrealised, promise. A validated model for illness stages 0 and 1 would provide the basis for developing preventive strategies and interventions which may delay the onset of BD and circumvent associated morbidity [10, 17], an approach which has shown promise in other serious mental disorders [18]. Identification of factors that arrest progression to the later, more debilitating stages of illness has potential to reduce loss of functioning, morbidity, mortality and treatment resistance [4, 8]. Developing validly stage-tailored interventions could ensure optimally effective psychological and pharmacological interventions are implicated for individuals at their specific illness stage [2, 7, 19]. Of particular relevance here, the stage of illness heuristic may also help clinicians and consumers come to a shared understanding of the most meaningful targets for interventions. For example, a focus on managing risk factors may benefit the asymptomatic at-risk group (in psychiatry, this group is only recognisable as those with a family history of a severe mental disorder), but low risk, non-pharmacological interventions would be most realistic for this group. Relief of symptoms and management of triggers may have greater impact for those at stages associated with more recent evolution of established illness (primarily stage 2), whereas the quality of life or functioning may be more meaningful intervention targets in stage 4 and for some in stage 3 [19, 20]. In later stages of illness, it is increasingly likely that combined psychopharmacological approaches will be employed, with increasing acknowledgement that psychological interventions are important in end-stage BD [21].

### Proxy measures of stage in established BD

In the absence of robust evidence for biomarkers and biobehavioural boundaries of the putative stages in BD, empirical research has relied on proxies to operationalise stage of illness. These have not been widely employed in subthreshold or asymptomatic stages of illness (i.e., stages 0 or 1), but have been increasingly considered in established BD. Most commonly, lifetime number of mood episodes has been used [22–27]. While a potentially useful and plausible proxy measure,^1^ number of episodes alone cannot fully capture the complexity of the proposed staging phenomenon [4, 9], nor the notion of ‘disease extension’, which is a fundamental component of medical staging models (e.g., a tumour spreads to lymph nodes, then to other bodily systems) [28]. It has been proposed that analogues of physical disease extension in the case of mental disorders may be comorbidities, functioning or disability, and cognitive functioning [2, 5, 9, 29]. For example, measures such as the Functional Assessment Short Test (FAST) or Social and Occupational Functioning Assessment Scale (SOFAS) differentiate between putative stages of established BD [5, 9, 29, 30]. Each of these plausible proxy measures has limitations, for example the circularity inherent in considering functioning at baseline as a predictor of functioning post-intervention and issues with equating number or type of comorbidities to severity or progression. Based on an extensive review of published models and theories of staging, the proxy measures for stage of illness recognised in this review, are outlined in Table 1.

**Table 1:** Plausible proxy measures for stage of illness relevant to this review

Variables	
1. Number of mood episodes [22–26], with consideration by episode type and of frequency where available	
2. Functioning/disability [5, 9, 29, 30]	
3. Cognitive functioning/cognitive impairment/neurocognitive deficit [2, 5, 9, 29]	
4. Comorbidities [9]	

Most research to date has relied upon the number of episodes as the proxy measure of stage of illness, while recognising that other clinical factors are relevant to stage of illness, and as such, these additional variables will be included as exploratory additional plausible proxy measures. Given the identified limitations of such proxies, all conclusions will consider these limitations and where available, change from premorbid functioning or cognition will be utilised. Other clinical course factors certainly have relevance to staging models, including age at onset, duration of illness, bipolar type and predominant polarity [16, 31]. However, due to lack of consistency in how these feature across staging proposals, this review will examine those potential proxy measures with most support to date.

### Psychosocial interventions and staging of illness

Research into staging of illness is in its infancy, with many questions remaining. Research to date suggests that both pharmacotherapy and adjunctive psychotherapy are more effective for some individuals with BD than for others, providing some support for the hypothesis that stage of illness may influence response to therapy or outcomes [19, 25, 32, 33]. However, evidence forwarded to support the assertion that stage of illness may moderate outcomes in BD rests almost entirely on post hoc statistical analyses of outcomes in clinical trials not designed to address the staging hypothesis.

An influential finding in this literature arose in post hoc analyses of a pragmatic effectiveness trial of cognitive-behaviour therapy (CBT) for BD [34] which demonstrated that CBT was superior to treatment as usual (TAU) alone in improving outcomes for individuals with 11 or fewer lifetime mood episodes, with no benefits of adding CBT to TAU in those with more than 11 episodes. Similar findings have been reported in several psychoeducation trials, wherein those retrospectively classified within earlier stages of established illness (using number of episodes or functioning as proxy measures) were more likely to report benefits than those retrospectively classified into later stages of illness [22, 30, 35, 36]. Findings from the psychotherapy arm of the large-scale Systematic Treatment Enhancement Program for Bipolar Disorder (STEP-BD) trial align with this; participants with fewer lifetime manic and depressive episodes were faster and more likely to respond to psychotherapy overall, especially lower intensity interventions [14, 37]. Extrapolating from studies to date is consistent with the staging hypothesis of BD, insomuch as psychological interventions may be generally less effective as BD advances.

There is growing recognition that non-clinical, recovery-focused outcomes are valued by consumers and that these are distinct from yet complementary to symptom-based intervention targets [20, 38]. These may include functional outcomes, such as social functioning including autonomy, occupational functioning, and interpersonal functioning and cognitive functioning including memory, learning and attention, and quality of life outcomes. For the present purposes, it is noteworthy that these outcomes have been argued to be particularly relevant to individuals with later stage established BD (stages 3–4): Berk and colleagues propose that as BD progresses, symptom remission may be a less important target for intervention, and recovery-focused outcomes such as improved quality of life and daily functioning may warrant higher priority [19].

A novel and complex hypothesis therefore emerges from this literature. As argued by Murray et al. [20], different outcomes may become more relevant as BD progresses, and different interventions may be best positioned to target these outcomes. For instance, Torrent and colleagues compared TAU pharmacotherapy with intensive psychoeducation and functional remediation in a sample of individuals with significant functional impairment and a large number of previous mood episodes, consistent with the later putative stages of BD [39, 40]. Participants in the functional remediation group reported greater improvements in functioning (particularly autonomy) than those receiving psychoeducation or TAU, and these differences reached significance at 12-month follow-up. Conversely, no significant differences in relapses or residual depressive symptoms were reported between groups at follow-up. These findings may suggest differential effectiveness of two interventions for those considered at the later stages of BD for improving functioning, but not symptoms. A recent pilot study of a mindfulness intervention targeted individuals with late-stage BD, based on number of episodes [32]. Moderate to large improvements were reported in participants’ quality of life, while symptom change was non-significant. While limited, this study encourages further research into the hypothesis that different outcomes may become relevant for individuals at later illness stages. Conversely, several psychoeducation trials with samples considered in the later stages of illness (based on functioning or number of episodes) have failed to find significant improvements in symptoms or recovery-focused outcomes, including quality of life and functioning. Taken together these findings may suggest that different interventions *and* different targets may be required as BD progresses [39–42].

Pharmacotherapy is the first-line treatment for bipolar disorder, with high-quality evidence supporting the effectiveness of medications for mania and depression in both the acute and maintenance phases (e.g., [43–45]). However, areas of uncertainty remain, such as effective options for addressing treatment resistance and refractory depression [45]. A number of systematic reviews and meta-analyses conducted in recent years support the effectiveness of psychosocial interventions as adjuncts to medication in the treatment of bipolar disorder [33, 46–48]. Overall, medium effect-size benefits have been found for outcomes including manic and depressive relapse, residual symptoms and medication adherence. All such reviews note significant limitations within the evidence base, including the small number of studies, heterogeneous assessment of outcomes, and limited study quality [49]. Several reviews have noted that factors relating to stage of illness (including the proxy measures examined in the proposed review) may partially moderate the effectiveness of these interventions [33, 47, 48], especially CBT and psychoeducation.

To the best of our knowledge, only two studies have directly reviewed plausible proxy measures of stage of illness as moderators of psychosocial interventions for BD. A meta-analysis of psychotherapy studies for BD aimed to assess whether number of lifetime mood episodes predicts response to psychotherapy [50]. This study did not find evidence for an impact of number of episodes in the effectiveness of psychological therapies for preventing relapse in BD. However, due to the methodological homogeneity required for meta-regression, only six studies were included, with the single outcome variable of relapse rates. This narrow scope excluded important findings in the literature, in particular those from the largest randomised controlled trial (RCT) of CBT for BD to date (above; excluded due to categorical recording of number of episodes) [34]. More recently, a narrative synthesis of the role of accumulating episodes was undertaken [25], concluding that response to psychotherapy declines with accumulating mood episodes. However, this review was confined to studies reporting on a direct comparison between those classified as being within early versus late stages of established BD; thus, only one RCT of group psychoeducation and one CBT trial were included. Furthermore, only symptom-focused outcomes (relapse rates and time to recurrence) were examined.

The limited number of outcomes examined in prior reviews has confined the exploration of the complexity and applications of staging of illness, disallowing conclusions to be drawn as to whether different outcomes may be more effective treatment foci at different stages of illness. Another key limitation of existing reviews is the use of a single plausible proxy measure, lifetime number of mood episodes [12, 51]. Further, both relied on post hoc analyses to draw conclusions, due to the dearth of studies explicitly seeking to explore stage of illness or plausible proxy measures thereof as a moderator of outcomes.

### The proposed systematic review

A comprehensive systematic review is now required which attends to (i) recovery-focused and functional outcome measures and (ii) an expanded range of plausible proxy measures of stage of illness in post-onset BD, derived from a combination of empirical data and prominent theoretical models. The proposed review will follow PRISMA guidelines and provide a synthesis of the evidence for stage-based moderation (as operationalised in plausible proxy-based moderation) of psychosocial interventions for recovery-focused outcomes in established BD and identify key priorities for further research.

The overarching aim of the planned review is to advance understanding of the notion of stage of illness in BD, by retrospectively interrogating findings related to psychosocial interventions’ impact on recovery-focused and functional outcomes in stages 2–4 of BD. Specifically, the objectives are to explore whether plausible proxy measures of stage of illness may moderate recovery-focused intervention outcomes in BD and whether there is any evidence that this is associated with specific types of intervention. It is expected that this review will answer two key questions regarding the status of the staging construct in BD: (i) is there any evidence that plausible proxy measures of stage moderate recovery-focused outcomes in adjunctive psychosocial treatment of established BD? (ii) If so, is the effect more strongly associated with particular interventions?

## Methods

The systematic review has been registered with the International Prospective Register of Systematic Reviews (PROSPERO, http://www.crd.york.ac.uk/PROSPERO, registration number: CRD42016037868). It will be conducted in accordance with Preferred Reporting Items for Systematic Reviews and Meta-Analyses Protocol (PRISMA-P) recommendations for systematic review protocols [52] (see Additional file 1), and the findings will be reported using the PRISMA guidelines [53].

### Criteria for study inclusion

#### Population

Included studies will be empirical and comprise participants aged 18 and over with a BD I, BD II, BD-NOS, not elsewhere classifiable, or other specified BD diagnosis (meeting internationally recognised diagnostic criteria as in the DSM-IV or 5, or ICD-10), without restrictions based on characteristics such as psychosis, comorbidity or mood status at study entry. Studies with participants under 18 years old have been excluded due to the additional complexity in diagnosis and treatment in this population, as well as potential divergent trajectory and phenomenology of early-onset or paediatric BD [54]. Studies with participants with other diagnoses (specifically schizophrenia and schizoaffective disorder) will be excluded unless these reports provide separate analyses relating to those with diagnosed BD.

#### Interventions

Studies will detail the outcomes of any psychological or psychosocial intervention or combination of psychosocial interventions (any non-medical intervention intended to modify symptoms, behaviour, emotional state, or feelings) for BD, including (but not restricted to) cognitive-behavioural therapy, mindfulness-based cognitive therapy, interpersonal and social rhythm therapy, psychoeducation, family-focused therapy, functional or cognitive remediation, schema therapy, acceptance and commitment therapy, dialectical behaviour therapy, and other mindfulness-based therapies. Intervention formats will include online, telephone and face to face, delivered individually or in a group (or combinations of these). Self-management interventions will be included, defined as any structured psychotherapeutic intervention accessed and completed wholly or partly by oneself, regardless of mode. For example, supported and unsupported online therapy programs, psychotherapeutic apps, and manualised therapies worked through independently will be included if they are formally assessed. Descriptions and details of all included interventions will be provided within the systematic review.

#### Comparisons

Studies must assess one or more psychosocial intervention/s alone or in combination. Descriptions and details of all included comparator interventions will be provided within the systematic review. Studies will not require a direct comparison between individuals at different putative stages of illness but must include analyses (or provide sufficient detail to draw this information from the study methodology and results) of a proxy measure of stage of illness.

#### Outcomes

For the present review, ‘recovery-focused’ primary outcomes will include outcomes in three categories: (i) general/social functioning (including functional impairment, daily general, social or occupational functioning, disability or autonomy), (ii) cognitive functioning or impairment and (iii) quality of life. Table 2 outlines the outcome measures to be included in this review.

**Table 2:** Outcome measures for the proposed review

Outcome measure instruments	
1. General/social functioning A multidimensional construct encompassing an individual’s capacity for independent living, occupational and educational achievement, interpersonal relationships and recreation [55]. Measures will include any validated self-report or clinician-rated, real-world functioning or performance-based measure, including the following: Global Assessment of Functioning (GAF) [56], Functioning Assessment Short Test (FAST) [57], The clinician-rated Longitudinal Interval Follow-up Evaluation-Range of Impaired Functioning Tool (LIFE-RIFT) [58], World Health Organisation Disability Assessment Schedule (WHODAS-2) [59], Sheehan Disability Scale (SDS) [60], Social Functioning Scale (SFS) [61], Life Functioning Questionnaire (LFQ) [62], USCD Performance-Based Skills Assessment [63], Bipolar Disorder Functioning Questionnaire [64], Social and Occupational Functioning Assessment Scale (SOFAS) [65]	
2. Cognitive functioning An individual’s range of abilities relating to cognition, memory and learning, including attention, executive function, verbal learning and memory, verbal fluency, processing speed, working memory, visual learning and memory, psychomotor speed, visuo-spatial ability) [66]. Measures will include any validated measure or screening tool assessing cognitive function or impairment overall or by domain including the following: Mini-Mental State Exam [67], Mini-Cog [68], Memory Impairment Screen (MIS) [69], General Practitioner Assessment of Cognition (GPCOG) [70], The clock drawing test (CDT) [71], Montreal Cognitive Assessment (MoCA) [72], FAST, cognitive domain [57], Weschler Adult Intelligence Scale (WAIS-IV) or its subtests [73], Wechsler Memory Scale (WMS-IV) or its subtests [74], Wide Range Assessment of Memory and Learning [75], California Verbal Learning Test [76], Hopkins Verbal Learning Test-Revised [77], Brief Visuospatial Memory Test-Revised [78], Rey-Osterrieth Complex Figure Test [79], Trail Making Test Part A or Part B [80], Wisconsin Card Sorting Test [81], Delis-Kaplan Executive Function System [82], Boston Naming Test [83], Controlled Oral Word Association [84], MATRICS Consensus Cognitive Battery (MCCB) [85]	
3. Quality of life While quality of life is used in varying ways, the World Health Organisation defines this as “an individual’s perception of their position in life in the context of the culture and value systems in which they live and in relation to their goals, expectations, standards and concerns” [86]. Measures will include any validated self-report or clinician-rated, general or disorder specific measure of quality of life, including the following: QoL.BD [87], EuroQoL [88], Lancashire Quality of Life Profile [89]/Manchester Short Assessment of Quality of Life (MANSA) [90], Lehman Quality of Life Interview [91], Longitudinal Interval Follow-up Evaluation [92], MOS Short Form 12[93],MOS Short Form 20 [94], MOS Short Form 36 [95], Quality of Life Enjoyment and Satisfaction Questionnaire (Q-LES-Q) [96], Quality of Life in Depression Scale [97], Quality of Life Index [98], World Health Organisation Quality of Life Assessment [99]	

#### Study type

RCTs and controlled trials will be sought. However, it is not anticipated that many RCTs will be available, especially for novel therapeutic modalities. Therefore, other intervention studies will be eligible for inclusion, including open pre-post trials and non-randomised controlled trials. Case series and case reports will be excluded due to the high risk of bias in these designs. Whilst broadening inclusion criteria invites the inclusion of studies with a higher risk of bias, this will be addressed with the implementation of risk of bias criteria and offset by the anticipated gains including the implications of review findings for future research and clinical practice.

### Procedures

#### Search strategy

Scopus, PsychInfo, PubMed and Web of Science databases will be searched to identify potentially eligible studies for inclusion. No date restrictions will be applied and search terms will be broad to increase the likelihood of capturing potentially eligible studies. Unpublished trials, ongoing trials and grey literature will not be included as these are not anticipated to contribute to the fulfilment of the review aims. An example of the proposed search strategy for Scopus is outlined in Table 3.

**Table 3:** Scopus search terms for the proposed review

Search terms	
TITLE-ABS-KEY (bipolar OR mania OR manic* OR “manic depress*”) AND TITLE-ABS-KEY (“psychological therap*” OR psychotherap* OR psychosocial OR “self-management” OR “Psychological intervention” OR cbt OR “cognitive therapy” OR “behavior therapy” OR “behavioural therapy” OR “cognitive behavioural” OR “cognitive behavior” OR mindfulness OR mbct OR “collaborative care” OR ipsrt OR “social rhythm” OR interpersonal OR “Acceptance and Commitment Therapy” OR “ACT” OR dbt OR “dialectical behaviour therapy” OR psychoeducation OR “family therapy” OR “family focused” OR carer OR “functional remediation” OR “cognitive remediation” OR “schema therapy”) AND TITLE-ABS-KEY (stage* OR staging OR predict* OR mediat* OR moderat* OR neurocognit* OR cognit* OR comorbid* OR episodes OR “number of episodes” OR function* OR trajectory OR “illness history” OR “illness characteristics” OR “course of illness”) AND TITLE-ABS-KEY (recovery OR “patient-reported outcomes” OR “quality of life” OR function* OR employment OR autonomy OR wellbeing OR social OR disability OR meaning OR vocation* OR cognit* OR acceptance OR self-compassion)	

#### Quality and risk of bias

Risk of bias within randomised trials will be assessed with the Cochrane Collaboration tool for assessing risk of bias [100], within non-randomised studies using the Risk Of Bias In Non-randomised Studies of Interventions (ROBINS-I) tool [101], and Grading of Recommendations Assessment, Development and Evaluation (GRADE) methodology across studies, with this assessment taken into consideration in any conclusions [102].

#### Data extraction and management

Following PRISMA-P guidelines, we will provide a flowchart to demonstrate each element of the search.

##### Relevance

After deduplication, both raters (HT and CM) will screen all titles and abstracts against inclusion criteria for relevance, specifically population, study type, interventions, and outcomes. The full text of results will be reviewed as necessary to ensure accurate categorisation. Uncertainties regarding eligibility for inclusion will be resolved via consensus. Where multiple publications of the same study or from the same dataset exist, these will be narrowed to a single summary that reports key variables relevant to this review.

##### Extraction

Data will be extracted independently by both raters from all included studies using a data extraction form developed for this review (Additional file 2). Both raters will pilot this form with five studies initially and make amendments if required prior to data extraction. Extracted data will include study design, sample size, study population, participant demographics (including diagnoses), baseline characteristics including details of stage-related proxy measures, details of the intervention, details of all reported study outcomes, and information required for assessment of the risk of bias. Where missing data are identified for variables relevant to this review, study authors will be contacted to request these. Studies will be excluded if authors do not provide these data.

#### Data synthesis

Data will be synthesised using narrative synthesis methods, as the broad aims and inclusion criteria will preclude meaningful statistical data pooling. A table of all the extracted data will be presented in the systematic review. A data-based convergent synthesis design will be employed [103], focusing on the interaction between interventions, stage of illness and outcomes. Participants’ likely stage of illness will be identified based on the plausible proxy measures and outcomes grouped into ‘cognitive’, ‘functioning’, and ‘quality of life’. Data-based convergent synthesis involves describing and synthesising results qualitatively, drawing out themes or categories. Data for each outcome type will be synthesised per putative stage of illness. Next, to address the second question, different intervention types will be examined.

## Discussion

This review will constitute a significant addition to the literature regarding the psychological treatment of BD, by applying the lens of ‘staging of illness’. Findings will further illuminate the role of plausible proxy measures of stage of illness in the effectiveness of psychological interventions for established BD, by synthesising the evidence regarding proxy measures of stage of illness moderating interventions for recovery-focused and functional outcomes.

There is a clear need for a summary of evidence relating to stage of illness in the treatment of established BD; however, reviews to date have focused on symptoms alone and a limited range of proxy measures for the staging construct [25, 50]. The current review extends previous reviews in several ways. Most importantly, the range of outcomes which may be meaningful for those with BD will be explored, including examination of their applicability across stages of illness. This is essential in light of previous findings suggesting that different outcomes may be relevant at different stages of illness [32, 39]. For example, symptom relief and relapse prevention appear less amenable to intervention with advancing illness [22, 30, 34] while quality of life and functioning have been effectively targeted later in the course of illness [32, 39]. Further, potential differences in effectiveness between interventions across putative stages of illness will be examined, as prior studies have suggested that particular intervention types have more relevance at different stages of illness [2, 22, 25, 32–34]. Broader eligibility criteria will enable the examination of other putative stage-related factors rather than relying solely on number of episodes as a proxy measure of stage of illness. Theoretically, a broader range of factors—such as functional impairment and neurocognitive functioning—may be relevant to stage of illness [29]. Such studies may provide new insights that were not available to prior reviews that were narrower in scope. If the review’s two driving questions are answered in the affirmative, the project will provide an empirical platform for the identification, development and tailoring of novel psychological intervention strategies for individuals with BD based on stage of illness, ultimately providing a more focused direction for clinicians, consumers and researchers.

However, it should be noted that qualitative systematic reviews have a number of limitations. These include often involving less rigour than quantitative data syntheses and being subject to researchers’ views and biases (e.g., [104]). We will attempt to minimise the impacts of these limitations through the rigorous methods described in this protocol. Another anticipated limitation is that few studies of any one intervention are expected, which may inhibit meaningful synthesis.

## Additional files

Additional file 1: PRISMA-P 2015 Checklist. (DOCX 34 kb)

Additional file 2: DATA EXTRACTION FORM. (DOCX 14 kb)

## Abbreviations

ACT: Acceptance and commitment therapy
BD I: Bipolar I disorder
BD II: Bipolar II disorder
BD: Bipolar disorder
BD-NOS: Bipolar disorder not otherwise specified
CBT: Cognitive-behavioural therapy
CM: Carla McEnery
DBT: Dialectical behaviour therapy
DSM: Diagnostic and Statistical Manual
FAST: Functioning Assessment Short Test
GAF: Global Assessment of Functioning
GM: Greg Murray
GRADE: Grading of Recommendations Assessment, Development and Evaluation
HT: Hailey Tremain
ICD: International, Classification of Diseases
IPSRT: Interpersonal and Social Rhythm Therapy
JS: Jan Scott
KF: Kathryn Fletcher
LFQ: Life Functioning Questionnaire
LIFE-RIFT: Longitudinal Interval Follow-up Evaluation-Range of Impaired Functioning Tool
MB: Michael Berk
MBCT: Mindfulness-based cognitive therapy
PRISMA: Preferred Reporting Items for Systematic Reviews and Meta-Analyses
PRISMA-P: Preferred Reporting Items for Systematic Reviews and Meta-Analyses Protocol
PROSPERO: Prospective Register of Systematic Reviews
RCT: Randomised controlled trial
ROBINS-I: Risk Of Bias In Non-randomised Studies of Interventions
SDS: Sheehan Disability Scale
SOFAS: Social and Occupational Functioning Assessment Scale
STEP-BD: Systematic Treatment Enhancement Program for Bipolar Disorder
TAU: Treatment as usual
WHODAS-2: World Health Organisation Disability Assessment Schedule

## Footnotes

1 We use the qualifier ‘plausible’ to remind the reader (and ourselves) that ‘staging’ remains a hypothetical construct in psychiatry. Any proxies are at best consistent with one or more of the various provisional conceptualisations of staging.
