# Supplementary material for: Does stage of illness influence recovery-focused outcomes after psychological treatment in bipolar disorder? A systematic review protocol
Source: Syst Rev. 2019 May 25;8:125. doi: 10.1186/s13643-019-1042-4 (PMC6535183; doi:10.1186/s13643-019-1042-4)
Supplement: Supplementary file 2 — DATA EXTRACTION FORM. (DOCX 14 kb) [file 13643_2019_1042_MOESM2_ESM.docx]

**Data extraction form**

**Reviewer initials ______________________**

| **Study details** | |
| --- | --- |
| **Study ID** | **Journal** |
| **First author surname, initial** | **Year of publication** |
| **Participants** | |
| **Population** | **Participant details: Sample size, age, duration of illness, medication, index episode** |
| **Intervention** | |
| **Intervention type (primary)** | **Intervention type (control)** |
| **Comparators** | |
| **Comparison:**    **YES NO** | **Comparator: (none/waitlist/active as above/placebo/TAU)** |
| **Outcomes** | |
| **Primary Outcome/s (incl. statistical results, CI, assessment measures)** | |
| **Secondary outcome/s (incl. statistical results, CI, assessment measures)** | |
| **Study design** | |
| **Design (RCT, pre-post control trial, crossover, cohort study, blinded?), follow up period.** | |
| **Staging information** | |
| **Proxy/ies of stage** | **How was this measured?** |
| **Data available for:**  **Baseline**  **Post-intervention**  **Follow-up** | **Proxy data notes** |
| **Risk of bias** | |
| **Selection bias: low, high, unclear** | **Randomisation? Allocation concealment?** |
| **Performance bias: low, high, unclear** | **Blinding (participant, staff)?** |
| **Detection bias**: **low, high, unclear** | **Blinding of outcome assessment?** |
| **Attrition bias: low, high, unclear** | **Intention to treat analysis? Numbers per group pre-post? Withdrawal/dropout reasons** |
| **Reporting bias: low, high, unclear** | **Selective reporting of outcomes?** |
